# Seroprevalence of IgG antibodies against hepatitis-A infection among individuals aged 6–30 years in India, 2021: a nationwide population-based cross-sectional study

**DOI:** 10.1016/j.lansea.2025.100669

**Published:** 2025-09-25

**Authors:** Muthusamy Santhosh Kumar, Chethrapilly Purushothaman Girish Kumar, Velusamy Saravanakumar, Thiyagarajan Karunakaran, Jeromie Wesley Vivian Thangaraj, Sriram Selvaraju, Kiran Rade, Ramasamy Sabarinathan, Surendran Parvathi, Smita Asthana, Rakesh Balachandar, Sampada Dipak Bangar, Avi Kumar Bansal, Jyothi Bhat, Debjit Chakraborty, Vishal Chopra, Dasarathi Das, Kangjam Rekha Devi, Gaurav Raj Dwivedi, S Muhammad Salim Khan, M Sunil Kumar, Avula Laxmaiah, Major Madhukar, Amarendra Mahapatra, Talluri Ramesh, Chethana Rangaraju, Jyotirmayee Turuk, Suresh Yadav, Tarun Bhatnagar, Balram Bhargava, Manoj V. Murhekar, ICMR serosurveillance group, Alka Turuk, Manjula Singh, Abdul Mabood Khan, Samiran Panda, Nivethitha N. Krishnan, Aby Robinson, Nivetha Srinivasan, Rushikesh Andhalkar, Anshuman Chaudhury, Prathiksha Giridharan, Vijayan Geetha Vinod Kumar, Naga Bhushanam Kotala, Krithikaa Sekar, Surabhi Yadav, Padmapriyadarshini Chandrasekaran, Anand Praveen Kumar, Vikas Dhikav, Ramesh Kumar Sangwan, Mahendra Thakor, Nimmathota Arlappa, Babu Jagjeevan, Hemalatha Rajkumar, Khalid Bashir, Inaamul Haq, Mariya Amin Qurieshi, Praveen K. Bharti, Pushpendra Singh, Debdutta Bhattacharya, Srikanta Kanungo, Jaya Singh Kshatri, Subrata Kumar Palo, Sanghamitra Pati, Rajni Kant, Hirawati Deval, Niraj Kumar, Rajeev Singh, Sthita Pragnya Behera, Ashok Kumar Pandey, Ashrafjit S. Chahal, Alok Kumar Deb, Shanta Dutta, Sarang Dhatrak, Ankit Viramgami, Amit Chakrabarti, Rakesh Dayal, Anindya Mitra, Arshad Kalliath, Anbarasi Kalaiselvan, Senthil Kumar Mayandi, Sreelakshmy Thalappil Naduvilethil, Neelima Devi Akula, Bhuvaneswari Anandhan, Sekar Dhanapriya Vadhani, Monisha Anbu, Pavithra Murugan, Prashant Kumar Singh, Shalini Singh, Hari Bhan Singh, Agam Jain, A.R. Nirmala, Seema Sahay, Kanwar Narain, Somashekar Narasimhaiah, Krishna Pandey

**Affiliations:** a ICMR National Institute of Epidemiology, Chennai, Tamil Nadu, India; b ICMR National Institute for Research in Tuberculosis, Chennai, Tamil Nadu, India; c WHO Country Office for India, New Delhi, India; d ICMR- National Institute of Cancer Prevention & Research, NOIDA, Uttar Pradesh, India; e ICMR-National Institute of Occupational Health, Ahmedabad, Gujarat, India; f ICMR-National Institute of Translational Virology and AIDS Research, Pune, Maharashtra, India; g ICMR-National JALMA Institute for Leprosy & Other Mycobacterial Diseases, Agra, Uttar Pradesh, India; h ICMR-National Institute of Research in Tribal Health, Jabalpur, Madhya Pradesh, India; i ICMR- National Institute for Research in Bacterial Infections, Kolkata, West Bengal, India; j State TB Training and Demonstration Centre, Patiala, Punjab, India; k ICMR- Regional Medical Research Centre, Bhubaneswar, Odisha, India; l ICMR- Regional Medical Research Centre, N.E. Region, Dibrugarh, Assam, India; m ICMR- Regional Medical Research Centre, Gorakhpur, Uttar Pradesh, India; n Govt Medical College Srinagar, Srinagar, Jammu and Kashmir, India; o State TB Training & Demonstration Centre, Thiruvananthapuram, Kerala, India; p ICMR - National Institute of Nutrition, Hyderabad, Telangana, India; q ICMR-Rajendra Memorial Research Institute of Medical Sciences, Patna, Bihar, India; r State TB Office, Hyderabad, Andhra Pradesh, India; s National Tuberculosis Institute, Bengaluru, Karnataka, India; t ICMR, National Institute for Implementation Research on Non-Communicable Diseases, Jodhpur, Rajasthan, India; u Indian Council of Medical Research, New Delhi, India

**Keywords:** Hepatitis A, Seroprevalence, IgG antibody, India, Vaccination

## Abstract

**Background:**

India accounts for one-fifth of the global hepatitis A virus (HAV) infections and half of HAV-related deaths. There is a lack of nationally representative population-based data on the endemicity of HAV to inform vaccination policy. We aimed to estimate the age-specific seroprevalence of HAV infection among individuals aged 6–30 years.

**Methods:**

We used serum samples collected during the fourth national COVID-19 serosurvey conducted between 14 June and 6 July 2021 to estimate the seroprevalence of HAV infection. The survey was conducted in 70 randomly selected districts across 20 Indian states and one union territory. We tested the serum samples from individuals aged six to 30 years for IgG antibodies against HAV. We estimated the overall and state-specific seroprevalence, along with 95% CIs, for the age groups of 6–10, 11–15 and 16–30 years. We classified the HAV endemicity in India using WHO classification (high, intermediate, low and very low).

**Findings:**

We tested 14,778 serum samples from individuals aged six to 30 years for IgG antibodies against HAV. Of these, 12,236 (90.0%, 95% CI 88.5–91.4) were found to be reactive. The seroprevalence increased with age, from 74.7% (71.1–77.9) among children aged 6–10 years to 85.2% (82.7–87.4) among those aged 11–15 years and 96.9% (96.3–97.5) among individuals aged 16–30 years. India was categorized as having intermediate endemicity for HAV infection as per the WHO classification. Of the 21 states or union territories included in the survey, 18 had intermediate endemicity.

**Interpretation:**

Our study findings indicate an intermediate level of endemicity for HAV infection in India. While these findings support consideration of hepatitis-A vaccination, further evidence on disease burden and cost-effectiveness is needed to inform policy decisions.

**Funding:**

10.13039/100000865Gates Foundation & 10.13039/501100001411Indian Council of Medical Research.

**Research in context**

**Evidence before this study**

We searched PubMed up to March 10, 2025, for estimates of seroprevalence of hepatitis A virus (HAV) infection in India using search terms “seroprevalence OR IgG antibodies” AND “hepatitis A” AND “India”. Of the 104 studies identified, 32 reported seroprevalence of HAV infection. We abstracted the age-group-specific seroprevalence and classified the studies into levels of endemicity based on WHO classification (high, intermediate, low and very low). Six studies reported high endemicity, 10 reported intermediate endemicity, and the remaining 16 studies did not cover the age groups required for classification. High endemicity was observed in studies conducted during the 1980s and 1990s, while studies from 2000 suggest a shift to intermediate endemicity. Most of these studies enrolled participants from tertiary care hospitals, had small sample sizes and lacked generalizability to different geographical regions in India, thereby limiting their utility in informing vaccination policy.

**Added value of this study**

In 2021, we conducted the fourth round of population-based serosurvey to estimate the prevalence of SARS-CoV-2 infection among individuals aged 6 years or more. We tested the serum samples from individuals aged six to 30 years from the fourth serosurvey for IgG antibodies against HAV infection. We found that about four-fifths of the Indian population aged 6–30 years had been exposed to HAV. The age-specific seroprevalence data indicated an intermediate level of endemicity in India. Of the 21 states or union territories studied, 18 states were classified as intermediate endemicity.

**Implications of all the available evidence**

Evidence on the seroprevalence of hepatitis A virus (HAV) suggests an intermediate level of endemicity in India, with variations observed in certain states. The study findings have implications for policy decisions regarding introducing HAV vaccination in the universal vaccination program.

## Introduction

Hepatitis A virus (HAV) is primarily transmitted through faecal-oral route, mainly due to faecal contamination of food or drinking water. HAV infections are common in low and middle-income countries (LMICs) with poor sanitary conditions. The virus can cause both sporadic infections and large-scale outbreaks. In LMICs, most children (90%) are infected with HAV by 10 years of age, through asymptomatic or mild infections. Hence, unexposed adolescents and adults could be at higher risk for symptomatic disease.^1^

In 2021, the Global Burden of Disease Study estimated that there were 160 million (95% CI: 152–170) HAV infections globally, leading to 26,901 deaths (95% CI: 18,387–42,454) and 1.8 million (95% CI: 1.3–2.8) disability-adjusted life years. India accounted for 19% of the global HAV infections, with an estimated 30.6 million cases (95%CI: 27.7–33.4) and 13,658 deaths (range: 8508–24,358), contributing to nearly half of the global HAV mortality.^2^

Several hospital-based studies across India have reported a high burden of symptomatic acute HAV infections confirmed by the presence of anti-HAV IgM antibodies among children and adolescents. Between 2014 and 2017, the Virus Research and Diagnostic Laboratory Network (VRDL) tested 24,000 specimens from suspected viral hepatitis cases and found that 3017 (12.6%) were HAV reactive, with 74.6% of these cases occurring among individuals aged below 19 years.^3^ Seven studies from tertiary care hospitals across India tested patients with acute viral hepatitis for IgM antibodies against HAV. Four studies included all age groups, two focused only on adults, and one included only children. The overall HAV IgM seropositivity ranged between 7.7% and 70.0%. These studies reported high seropositivity among children, adolescents and adults less than 30 years of age.^4^, ^5^, ^6^, ^7^, ^8^, ^9^, ^10^ (Appendix p 3) A review of published HAV outbreaks from India also revealed a high attack rate among children and adolescents, except in Kerala, where the attack rates were higher among adults (Appendix p 4–5). These findings suggest a shift in the epidemiology of HAV infection in India, with increasing symptomatic infections in adolescents and adults.

WHO recommends estimating age-specific seroprevalence to describe the epidemiological situation of HAV infection in a country. Such studies can provide information on susceptibility to infection in each age group and monitor the shift in risk of infection from childhood to older age groups. Using age-specific seroprevalence data, the levels of endemicity in a geographical area could be classified as high, intermediate, low and very low. A shift from high to intermediate endemicity is one of the criteria for deciding on the introduction of vaccination against HAV in the national immunization schedule.^11^ In India, vaccination against HAV is not yet included in the universal immunization program (UIP). HAV vaccine is available in the private sector. According to National Family Health Survey-5 (NFHS-5) data, only 4.2% of children aged 12–23 months received the UIP vaccines from the private sector.^12^

Although many seroepidemiological studies have been conducted in India in the past (Appendix p 6–11), they had certain limitations, including restricted geographical coverage, health facility-based recruitment of study participants, small sample sizes, and using laboratory assays with varying sensitivity and specificity. Moreover, some of these studies used different age groups, making it difficult to classify the areas according to the levels of endemicity as per the WHO classification. Hence, there is a need for a nationally representative study with a sufficiently large sample size, covering wide geographic areas and using standard serological assays to classify the geographical areas by levels of endemicity for HAV infection.^13^

The Indian Council of Medical Research (ICMR) conducted four rounds of nationally representative serosurveys during 2020–21 to estimate the prevalence of IgG antibodies against SARS-CoV-2 infection. The first survey was done among adults, the second and third serosurveys were done among individuals aged 10 years or more, and the fourth survey was done among individuals aged 6 years or more. We tested the fourth serosurvey samples from individuals aged from six years to 30 years to estimate the age-specific seroprevalence of HAV infection in India. The primary objective of the study was to classify the level of HAV endemicity at the national level using WHO criteria.^11^ As a secondary objective, we also aimed to assess endemicity at the state level.

## Methods

### Study design and participants

The methods for the fourth serosurvey to estimate the prevalence of IgG antibodies against SARS-CoV-2 infection are described elsewhere.^14^ Briefly, the serosurvey was conducted between 14 June and 6 July 2021, across 70 randomly selected districts in 20 states and one union territory in India. The survey used a multi-stage cluster sampling approach. From each district, we randomly selected 10 clusters (villages in rural areas and wards in urban areas) using the probability proportional to the population size method. Within each cluster, four random starting points were chosen, and 10 consenting individuals (one child aged 6–9 years, two individuals aged 10–17 years, and seven adults aged ≥ 18 years) were enrolled from each of the selected starting points. The number of individuals in the three age groups was based on the age structure of India’s population. The assumptions for sample size calculation of the first COVID-19 serosurvey are provided in the appendix (Appendix p 12). All sera were stored at −80 °C at ICMR-National Institute of Epidemiology, Chennai. We included samples from individuals aged six to 30 years to estimate the seroprevalence against HAV infection.

The Institutional Human Ethics committees of ICMR-National Institute of Epidemiology approved the study protocol for testing residual samples for IgG antibodies against hepatitis A (Ethics committee approval number NIE/IHEC/202308-06). During the fourth national serosurvey for COVID-19, we obtained written informed consent for participation in the survey as well as to preserve leftover sera samples for research studies, from those aged more than 18 years, written assent and written consent from parents for children aged seven to 17 years, and parental consent for children aged six years.

### Procedures

We tested the serum samples using the ARCHITECT HAVAb-IgG assay (Abbott) for the presence of IgG anti-HAV. The presence of IgG antibodies indicates either a past infection or vaccination against HAV. The presence of IgG anti-HAV in the specimen was determined by comparing the chemiluminescent signal in the reaction (measured as relative light units (RLUs)) to the cutoff signal determined from an active calibration. Specimens with signal to cutoff (S/CO) values ≥ 1.00 are considered reactive, whereas S/CO values < 1.00 are non-reactive. The assay has a sensitivity of ≥98% and specificity of ≥99.2% (95% CI: 98.4%–99.6%). We randomly selected five percent of samples and retested them for quality control.

### Statistical analysis

We described the characteristics of study participants as percentages, means, and standard deviations. The data were analyzed to estimate the weighted seroprevalence of IgG antibodies and 95% confidence intervals (CIs). National-level seroprevalence estimates were generated for three age groups: 6–10, 11–15, and 16–30 years, and also for gender and place of residence. To estimate the national seroprevalence, we used binary logistic random-effects models with random intercepts to account for clustering at the village or ward and household levels, and adjusted for design weights. The model with two levels (cluster & households) was statistically significant as compared to model with one level (cluster) (P-value ≤ 0.001). Details of the design weight calculation are given in Appendix p 12. The lme4 package in R software was used for the analysis. To estimate the age group-specific seroprevalence across different states, we used the complex survey analysis module in STATA, accounting for clustered data and applying normalized design weights.

We classified the HAV endemicity at the national (primary objective) and state level (secondary objective) using age-specific seroprevalence. We used the WHO classification for endemicity: 1) high (seroprevalence of ≥90% in the age group of 6–10 years) 2) intermediate (≥50% by 15 years of age with <90% by age 10 years) 3) low (≥50% by age 30 years with <50% by age 15 years) 4) very low (<50% by age 30 years).^11^ We constructed an age-specific seroprevalence curve using a generalized additive model. We assessed the agreement between the initial and repeat test results of the samples selected as part of quality control using the intraclass correlation coefficient. All the data analysis was done using STATA version 16 and R.

### Role of the funding source

The study was funded by the Gates Foundation, which had no role in the study design, data collection, data analysis, data interpretation, or writing of the report.

## Results

**Fig. 1:**
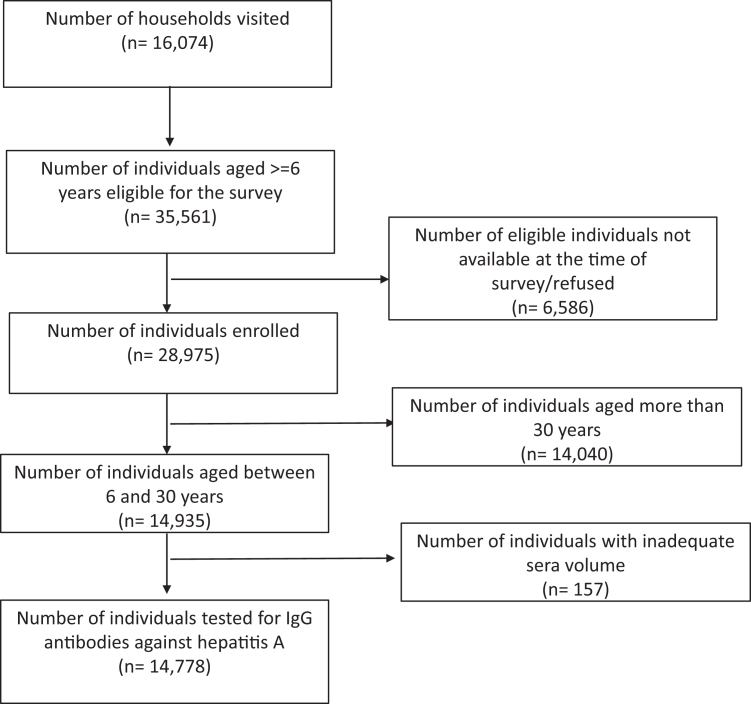
Flowchart describing enrollment of study participants.

The survey teams visited 16,074 households and identified 35,561 individuals aged six years or older. We collected blood samples from 28,975 individuals after obtaining consent, of whom 14,935 were aged between six and 30 years. Serum samples from 14,778 individuals with sufficient volume were tested for IgG antibodies against HAV (Fig. 1). Among those tested, 3266 (22.1%) were aged 6–10 years, 3357 (22.7%) were aged 11–15 years, and 8155 (55.2%) were aged 16–30 years (Table 1). The median age of the study population was 16 years [interquartile range (IQR) 11–23 years].

**Table 1:** Participant characteristics.

Characteristics	N = 14,778 n (%)
**Age in years**	
6–10	3266 (22.1)
11–15	3357 (22.7)
16–30	8155 (55.2)
**Gender**	
Male	7267 (49.2)
Female	7492 (50.7)
Others	19 (0.13)
**Area of residence**	
Rural	11,230 (75.9)
Urban slum	934 (6.3)
Urban non-slum	2614 (17.7)

**Fig. 2:**
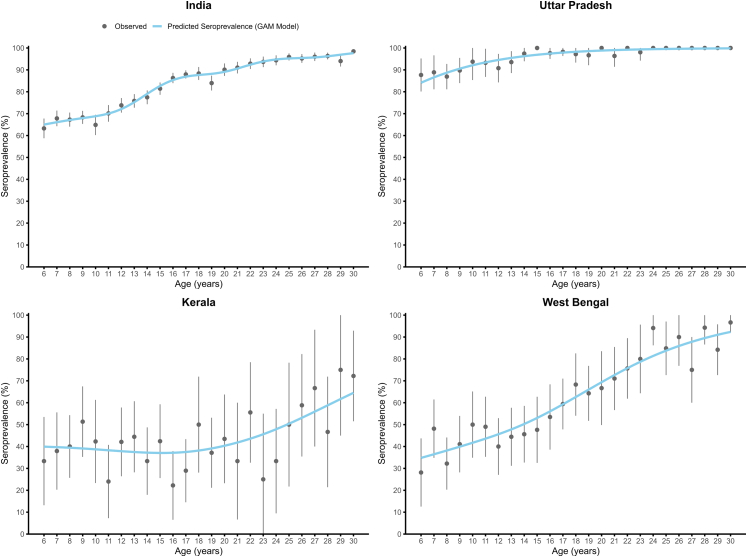
Age-specific seroprevalence curve.

Of the 14,778 sera tested, 12,236 (90.0%, 95% CI: 88.5–91.4) had IgG antibodies against HAV. The seroprevalence increased from 74.7% (71.1–77.9) among children aged 6–10 years to 85.2% (82.7–87.4) among children aged 11–15 years and 96.9% (96.3–97.5) among individuals aged 16–30 years. Age-specific seroprevalence curves indicate increasing seroprevalence with age (Fig. 2). (Appendix p 14–18) Based on the WHO criteria, India was classified as having an intermediate endemicity level for HAV infection. The seroprevalence was not different by sex. Individuals residing in urban slums had a slightly higher seroprevalence (91.8%, 86.1–95.3) compared to those living in urban non-slum areas (85.4%, 79.9–89.6), though the difference was not significant (P value = 0.282) (Table 2).

**Table 2:** Seroprevalence by gender and residence in different age groups, 2021.

Characteristics	6–10 years			11–15 years			16–30 years			Total		
	Tested	Positive	Weighted % (95% CI)	Tested	Positive	Weighted % (95% CI)	Tested	Positive	Weighted % (95% CI)	Tested	Positive	Weighted % (95% CI)
**Gender**												
Male	1746	1191	76.2 (70.9–80.7)	1706	1315	88.3 (84.9–91.0)	3815	3476	96.9 (95.8–97.7)	7267	5982	89.0 (87.2–90.5)
Female	1518	991	79.0 (74.2–83.2)	1646	1233	86.5 (82.8–89.5)	4328	4014	97.9 (97.1–98.4)	7492	6238	91.1 (89.6–92.4)
**Residence**												
Rural	2426	1657	78.0 (73.1–82.1)	2487	1920	87.5 (84.1–90.2)	6317	5848	97.4 (96.6–98.1)	11,230	9425	90.5 (88.9–91.9)
Non-slum	611	374	73.9 (61.2–83.6)	640	444	82.5 (70.8–90.1)	1363	1197	95.6 (92.0–97.7)	2614	2015	85.4 (79.9–89.6)
Urban slum	229	152	77.2 (60.9–88.1)	230	188	93.8 (84.7–97.7)	475	456	98.8 (96.2–99.6)	934	796	91.8 (86.1–95.3)

**Fig. 3:**
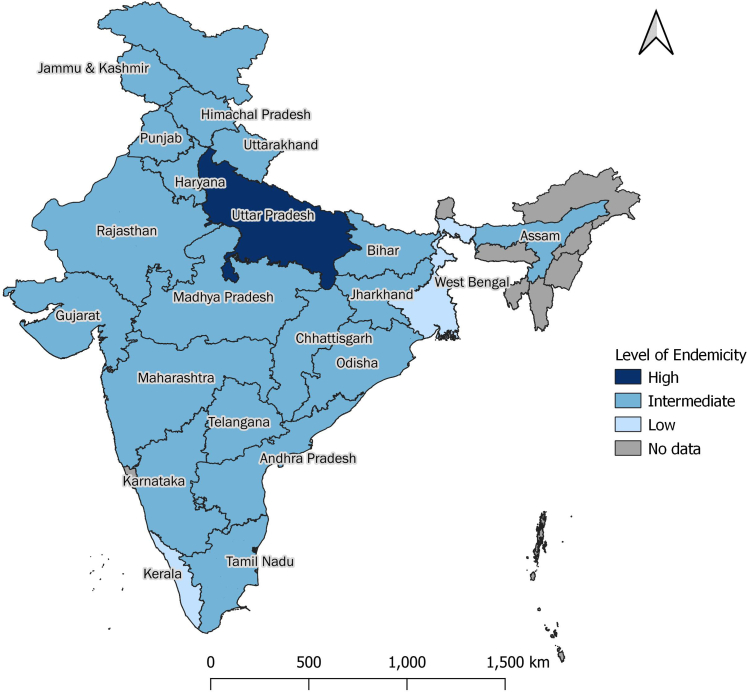
Endemicity of hepatitis A virus infection in India by states.

Among the 21 states included in the study, Uttar Pradesh had the highest overall seroprevalence (97.0%, 95.7–98.0), with prevalence exceeding 90% in all age groups, consistent with high endemicity. Kerala had the lowest overall seroprevalence at 44.8% (38.0–51.9). Age-specific seroprevalence in Kerala was 40.5% (35.2–45.9) in children aged 6–10 years, 40.0% (33.4–47.0) in those aged 11–15 years, and 51.1% (37.7–64.3) in individuals aged 16–30 years, indicating low endemicity. In West Bengal, the seroprevalence increased with age—from 42.5% (30.6–55.4) in children aged 6–10 years to 44.2% (29.9–59.5) in those aged 11–15 years, and 78.6% (72.2–83.8) in individuals aged 16–30 years—also suggesting low endemicity. However, the upper limit of the confidence interval for seroprevalence in age groups of 6–10 and 11–15 years exceeded the 50% threshold used in the WHO classification, implying uncertainty in endemicity classification (Table 3). All remaining states had seroprevalence patterns consistent with intermediate endemicity (Fig. 3).

**Table 3:** Seroprevalence of IgG antibodies against hepatitis A virus infection in India by age and state, 2021.

State	6–10 years			11–15 years			16–30 years			Total			Endemicity
	Tested	Positive	Weighted % (95% CI)	Tested	Positive	Weighted % (95% CI)	Tested	Positive	Weighted % (95% CI)	Tested	Positive	Weighted % (95% CI)	
Andhra Pradesh	158	104	54.4 (39.6–68.4)	173	132	73.8 (60.3–83.9)	341	321	86.3 (73.0–93.6)	672	557	76.7 (68.3–83.4)	Intermediate
Assam	124	95	80.1 (67.4–88.7)	120	105	83.7 (62.8–94.0)	346	322	92.6 (88.2–95.5)	590	522	88.5 (83.7–92.1)	Intermediate
Bihar	287	269	87.7 (74.3–94.6)	293	285	98.2 (95.0–99.4)	690	685	99.6 (98.9–99.9)	1270	1239	96.1 (89.1–98.7)	Intermediate
Chhattisgarh	122	98	76.2 (58.3–88.0)	107	87	79.3 (60.9–90.4)	431	409	92.4 (87.0–95.7)	660	594	86.9 (80.7–91.3)	Intermediate
Gujarat	117	66	55.1 (41.5–68.0)	103	66	65.4 (51.9–76.8)	423	383	91.2 (83.9–95.4)	643	515	81.0 (73.7–86.6)	Intermediate
Haryana	44	29	74.3 (62.9–83.2)	48	35	67.8 (56.5–77.4)	95	91	93.7 (89.1–96.5)	187	155	82.8 (73.2–89.5)	Intermediate
Himachal Pradesh	45	19	43.0 (26.4–61.3)	49	34	68.6 (49.5–83.0)	84	70	85.1 (71.6–92.8)	178	123	70.8 (56.2–82.0)	Intermediate
Jammu & Kashmir	43	21	40.4 (28.1–54.0)	42	28	59.6 (44.6–72.9)	112	107	96.2 (87.5–98.9)	197	156	74.6 (57.2–86.6)	Intermediate
Jharkhand	131	114	87.5 (72.0–95.0)	121	110	92.8 (85.6–96.6)	377	367	99.0 (97.0–99.7)	629	591	95.2 (91.0–97.5)	Intermediate
Karnataka	175	103	53.5 (39.1–67.4)	180	146	82.9 (73.4–89.5)	384	362	96.8 (93.6–98.4)	739	611	83.1 (70.2–91.1)	Intermediate
Kerala	158	66	40.5 (35.2–45.9)	168	64	40.0 (33.4–47.0)	278	121	51.1 (37.7–64.3)	604	251	44.8 (38.0–51.9)	Low
Madhya Pradesh	117	72	69.0 (53.2–81.4)	153	123	84.9 (74.5–91.5)	357	353	99.3 (97.7–99.8)	627	548	90.7 (85.2–94.2)	Intermediate
Maharashtra	283	204	75.9 (65.6–83.8)	316	263	86.3 (78.9–91.4)	649	634	98.1 (95.9–99.1)	1248	1101	90.4 (86.7–93.1)	Intermediate
Odisha	141	104	75.2 (66.6–82.1)	132	108	83.3 (72.3–90.5)	335	321	95.5 (91.0–97.8)	608	533	87.9 (82.9–91.5)	Intermediate
Punjab	171	90	49.1 (40.1–58.2)	171	111	55.0 (41.5–67.8)	372	327	88.5 (82.3–92.7)	714	528	69.7 (60.4–77.6)	Intermediate
Rajasthan	149	94	63.3 (48.5–76.0)	142	112	79.3 (65.4–88.6)	422	409	96.1 (91.6–98.2)	713	615	86.0 (79.6–90.5)	Intermediate
Tamil Nadu	143	82	55.3 (43.8–66.3)	139	94	72.7 (59.2–83.0)	260	228	88.3 (80.7–93.1)	542	404	75.1 (67.1–81.7)	Intermediate
Telangana	165	76	51.1 (36.0–66.0)	188	123	68.9 (53.2–81.1)	379	333	87.4 (75.9–93.9)	732	532	74.8 (66.2–81.8)	Intermediate
Uttar Pradesh	405	359	91.0 (85.8–94.5)	403	384	97.2 (94.5–98.6)	1149	1136	99.2 (98.6–99.6)	1957	1879	97.0 (95.7–98.0)	High
Uttarakhand	45	20	37.8 (15.6–66.6)	50	25	60.7 (30.6–84.4)	80	74	95.1 (83.8–98.7)	175	119	70.6 (50.2–85.1)	Intermediate
West Bengal	243	98	42.5 (30.6–55.4)	259	117	44.2 (29.9–59.5)	591	448	78.6 (72.2–83.8)	1093	663	63.1 (55.0–70.5)	Low
Overall	3266	2183	74.7 (71.1–77.9)	3357	2552	85.2 (82.7–87.4)	8155	7501	96.9 (96.3–97.5)	14,778	12,236	90.0 (88.5–91.4)	Intermediate

For quality control, we randomly selected and retested 746 sera samples using the same ARCHITECT HAVAb-IgG assay (Abbott). We found that 96.8% of the results were concordant. The intraclass correlation coefficient was 0.91 (0.89–0.92), indicating good agreement between the initial and repeat test results (Appendix p 13).

## Discussion

Our study found that about four-fifths of the Indian population aged 6–30 years were exposed to HAV. Seroprevalence increased with age, and age-specific seroprevalence data indicated an intermediate level of endemicity at the national level. The observed age group-specific seroprevalence patterns were suggestive of an intermediate level of endemicity in 18 of the 21 states and low endemicity in Kerala.

In 2008, WHO systematically reviewed the global seroprevalence of HAV infection, which included 28 studies from India. Most of these studies, conducted between 2001 and 2008, reported seroprevalence exceeding 90% among adolescents and adults, though lower levels were observed in high-income urban areas.^15^ Our age group-specific seroprevalence estimates indicated intermediate endemicity in India. While interpreting seroprevalence data can be challenging in countries with high HAV vaccine coverage, this concern is less relevant in India, where the HAV vaccine is not included in the UIP and only a small proportion (<5%) of children receive vaccines given in the childhood immunization program, through the private sector.^12^ Many recent studies in India also support a shift from high to intermediate endemicity.^6^^,^^16^

Our findings indicate low endemicity in the state of Kerala, reflecting changing HAV epidemiology driven by improved living conditions. The state of Kerala has the highest human development index (HDI) score (0.775) in India and the highest proportion of households with access to a toilet facility in the country. Sustained investments in public health, sanitation, education, and healthcare access may have contributed to lower early-life HAV exposure (Appendix p 20). These findings are further supported by the evidence from HAV outbreaks in the state, with high attack rates among adults indicating lower childhood exposure (Appendix p 4–5). A serosurvey conducted in a village in Kerala during a 2016 outbreak reported a seroprevalence of 49.7% among adolescents and adults. Of the 27 children under nine years tested for IgG against HAV, only three were reactive, suggesting low endemicity of infection.^17^

Our data suggested a low endemicity in West Bengal. A multi-centric hospital-based study conducted in 2019 reported low endemicity in Kolkata with a seroprevalence of 35.7% among children aged six to 10 years, 43.4% in those aged 11–15 years and 61.9% in 16–25 years, indicating a low endemicity.^16^ However, the wide confidence intervals for age-specific seroprevalence in our study—particularly for the 6–10 and 11–15-year age groups, where the CI crossed the 50% threshold—suggest uncertainty in the endemicity classification, likely due to a small sample size. Additional studies with larger sample sizes are needed to accurately determine the endemicity status in West Bengal.

In contrast, Uttar Pradesh showed high endemicity, with HAV seroprevalence exceeding 90% in all three age groups. Another single hospital study from Lucknow reported a seroprevalence of 73% and 83% among children in the first and second decades of life, respectively, suggesting intermediate endemicity.^18^ However, sample sizes were small (52 and 77 respectively), whereas our study included children from nine districts in the state with an adequate sample size to estimate age-specific seroprevalence more precisely. The high seroprevalence observed in Uttar Pradesh reflects ongoing exposure to HAV due to poor sanitation and lower socio-economic conditions. The state of Uttar Pradesh had a low HDI score (0.592) and a low proportion of households with access to toilets^19^ (Appendix p 20).

WHO recommends considering the introduction of hepatitis A vaccination for individuals aged 12 months or older based on the following criteria: transition from high to intermediate endemic level; an increasing trend of acute hepatitis, including severe disease among older children, adolescents or adults; and evidence of cost-effectiveness.^11^ Our survey findings indicate that most Indian states have intermediate endemicity of infection. We identified six studies in which patients with acute liver failure were tested for anti-HAV IgM and other hepatotropic viruses. The studies reported that HAV infection accounted for 21.4%–65.9% of acute liver failure cases. All the studies highlighted that HAV as one of the commonest causes of acute liver failure in both children and adults.^20^, ^21^, ^22^, ^23^, ^24^, ^25^ (Appendix p 19) However, these studies were limited to a few major cities, and there is a lack of data on the burden of symptomatic and severe disease across diverse geographic regions in India. There is a need for research to address this gap by generating evidence on the burden of symptomatic and severe HAV disease at the national level through existing surveillance systems and hospital-based data sources.^26^

Cost-effectiveness is the third criterion for introducing HAV vaccine into the immunization program. A systematic review of 43 economic evaluation studies on the HAV vaccination, including 15 from middle-income countries, found that universal childhood vaccination without screening was cost-effective. However, the review noted a lack of studies from India.^27^ A recent study conducted in Kerala evaluated the cost-effectiveness of various HAV vaccination strategies compared to the no vaccination scenario. The findings indicated that vaccination was cost-saving for both one-year-old children and 15-year-old individuals, regardless of whether serological screening was used prior to vaccination.^28^ There is a need for national-level economic evaluation studies in India to inform and guide the vaccination policy.

Our study has a few limitations. First, we did not include children under six years of age. However, seroprevalence among children aged 6–30 years was sufficient to classify HAV endemicity in line with WHO guidelines. Second, the sample sizes for estimating state-level prevalence—our secondary objective—may not have been sufficient, particularly for smaller states and in age groups with lower prevalence, such as children aged 6–10 and 11–15 years, to precisely estimate age-specific seroprevalence. Third, although the age and gender distribution of our sample was comparable to the 2011 census data, the proportion of participants from rural areas was higher (76.0%) than reported in the census (68.8%) (Appendix p 21). Fourth, we did not test samples for IgM antibodies, which could have provided insights into recent or ongoing HAV transmission. Future studies could include testing for both IgG and IgM antibodies to have a more comprehensive understanding of HAV epidemiology, particularly in the 6 to 10-year age group. Lastly, we could not explore factors associated with seropositivity in our analysis due to limited metadata.

In conclusion, our study findings indicate an intermediate level of endemicity for HAV infection in India. Our findings also suggest an intermediate level of endemicity in most Indian states, with high endemicity in Uttar Pradesh and low endemicity in Kerala. While this supports consideration of hepatitis-A vaccination, further data on disease burden and cost-effectiveness are needed to guide evidence-based policy decisions.

## Contributors

MVM, TB, BB, KR, MSK, JSVT, SS, VS, and RS designed and coordinated the fourth COVID-19 serosurvey. MSK and MVM developed the proposal on hepatitis A serology and acquired funding. SA, RB, SDB, AKB, JB, DC, VC, DD, KRD, GRD, AJ, SMSK, MSK, AL, MM, AM, TR, CR, JT, and SY led the data and blood sample collection with support from the authors listed in the ICMR serosurveillance group. VS, SP, RS, MSK and MVM accessed and verified the data. RS did data management. CPG and TK coordinated the laboratory testing. VS and SP did the data analysis. MSK and MVM wrote the first draft of the manuscript. All authors reviewed and approved the final draft of the manuscript.

## Data sharing statement

The de-identified datasets with the data dictionary are given as a Supplementary appendix along with this manuscript.

## Declaration of interests

We declare no competing interests.

## Appendix A Supplementary data

HAV_Seroprevalence_Dataset
