# Supplementary material for: Seroprevalence of IgG antibodies against hepatitis-A infection among individuals aged 6–30 years in India, 2021: a nationwide population-based cross-sectional study
Source: Lancet Reg Health Southeast Asia. 2025 Sep 25;41:100669. doi: 10.1016/j.lansea.2025.100669 (PMC12506519; doi:10.1016/j.lansea.2025.100669)
Supplement: Supplementary Tables 1–8 [file mmc2.docx]

**SUPPLEMENTARY APPENDIX**

SUPPLEMENT TO:

Seroprevalence of IgG antibodies against hepatitis-A infection in India, 2021: A population-based cross-sectional study

**Table of Contents Page No.**

1. Table 1: Hospital-based studies on symptomatic acute viral hepatitis (AVH) patients tested for IgM antibodies against hepatitis A in India………………………………………………………...………….3
2. Table 2: Profile of hepatitis A outbreaks reported in literature from India ..……………….....….……4-5
3. Table 3: Profile of studies on seroprevalence of IgG antibodies against HAV in India…….……...….6-9
4. Table 4: Results from studies on seroprevalence of IgG antibodies against HAV in India .......…....10-11
5. Supplementary methods: Sample size and sampling methods of COVID-19 serosurvey…………...…12
6. Table 5: Quality assurance of laboratory testing …..…………………………….………………..……13
7. Figure: Age-specific seroprevalence curve by state……………………………………….………..14-18
8. Table 6: Findings from studies that tested Acute Liver Failure cases for IgM antibodies against hepatitis A virus in India………………………………………………………………………………………….19
9. Table 7: National Family Health Survey data on sanitation and Human Development Index (HDI) score………………………………………………………………………………………………….…20
10. Table 8: Comparison of demographic characteristics of the study population with the 2011 census data……………………………………………………………………………………………………...21
11. STROBE Checklist……....………………………………………………………………………..……22

**Table 1:** **Hospital-based studies on symptomatic acute viral hepatitis (AVH) patients tested for IgM antibodies against hepatitis A in India**

| **R.No** | **Author/Year of publication** | **Age group of AVH cases** | **Location** | **# Acute viral hepatitis cases included (N)** | **# positive for HAV (n)** | **% positive for HAV (n/N)*100** | **Remarks** |
| --- | --- | --- | --- | --- | --- | --- | --- |
| 1 | Anitha T 2025 | All ages | Thanjavur, Tamil Nadu | 656 | 142 | 21.6 | High positivity in children and adolescents |
| 2 | Grover M 2024 | All ages | New Delhi | 14,807 | 1144 | 7.7 | Median age of HAV cases 16 years (IQR: 9-21) |
| 3 | Jain M 2024 | Adults | Indore, Madhya Pradesh | 513 | 155 | 30.2 | Median age of HAV cases 24 (18-35) years. 90% of cases in 18-30 years |
| 4 | Sood V 2019 | Children <18 years | New Delhi | 958 | 431 | 44.9 | Median (IQR) age of the patients was 11 yrs (6-14) |
| 5 | Talukdar AJ 2016 | Adults | Gauhati, Assam | 120 | 84 | 70.0 | 6 of 84 cases had fulminant hepatic failure |
| 6 | Joon A 2014 | All ages | Mangalore, Karnataka | 958 | 160 | 16.7 | Highest positivity in 21-25 years |
| 7 | Jain P 2013 | All ages | Lucknow, Uttar Pradesh | Children: 97  Adults: 108 | Children: 33  Adults: 31 | Children: 34.0  Adults: 28.7 |  |

1. Anitha T, Sivagamasundari P, Jacob ES, Kumar PBP. A Retrospective Study on the Prevalence of Hepatitis A Virus in Patients With Acute Viral Hepatitis at a Tertiary Care Hospital. Cureus. 2025 Feb 7;17(2):e78676.
2. Grover M, Gupta E, Samal J, et al. Rising trend of symptomatic infections due to Hepatitis A virus infection in adolescent and adult age group: An observational study from a tertiary care liver institute in India. Indian J Med Microbiol. 2024 Jul-Aug;50:100653.
3. Jain M. Rising incidence of acute viral hepatitis A in young adults- A cause for concern! Indian J Gastroenterol. 2025 Apr;44(2):259-260.
4. Sood V, Lal BB, Gupta E, et al. Hepatitis A Virus-related Pediatric Liver Disease Burden and its Significance in the Indian Subcontinent. Indian Pediatr. 2019 Sep 15;56(9):741-744.
5. Talukdar AJ, Islam S, Kashyap P, Dutta S. Sporadic adult acute viral hepatitis in Northeast India is predominantly hepatitis A virus related. Indian J Gastroenterol. 2016 Sep;35(5):401-402.
6. Joon A, Rao P, Shenoy SM, Baliga S. Prevalence of Hepatitis A virus (HAV) and Hepatitis E virus (HEV) in the patients presenting with acute viral hepatitis. Indian J Med Microbiol. 2015 Feb;33 Suppl:102-5.
7. Jain P, Prakash S, Gupta S, et al. Prevalence of hepatitis A virus, hepatitis B virus, hepatitis C virus, hepatitis D virus and hepatitis E virus as causes of acute viral hepatitis in North India: a hospital based study. Indian J Med Microbiol. 2013 Jul-Sep;31(3):261-5.

**Table 2:** **Hepatitis A outbreaks reported in the literature from India**

| **R.No** | **Author/year of publication** | **Year of investigation** | **Location** | **Residence** | **# cases** | **Age details of the cases** |
| --- | --- | --- | --- | --- | --- | --- |
| 1 | Joshi YK, 1985 | 1980 | Kochi, Kerala | Urban | 376 | 362 children and 14 adults |
| 2 | Chadha MS 2009 | 2004 | Daund, Pune, Maharashtra | Urban | 179 | >5 years: 44.5%; Highest attack rate in 6-10 years: (65.8%)  11-15 years: Similar to the 6-10 years age group |
| 3 | Arankalle VA, 2006 | 2005 | Kottayam, Kerala | Medical college | 540 | 18-20 years: 17/40 (42.5%)  21-25 years: 29/70 (41.4%)  26-35 years: 2/10 (20%)  36-52 years: 2/8 (25%) |
| 4 | Chobe LP 2009 | 2007 | Shimla,  Himachal Pradesh | Urban | 55 | Age range:2.5 to 25 years # cases by age  <10 years: 17  11-20 years:30  21-25 years: 8 |
| 5 | Rakesh PS 2014 | 2013 | Mylapore, Kollam, Kerala | Rural | 45 | <5 years: 3/235 (1.3%)  5-14 years: 15/480 (3.1%)  15-24 years: 22/478 (4.6%)  >25 years: 5/1910 (0.3%) |
| 6 | Pal S 2016 | 2013 | Rudraprayag, Uttarakhand | Flood camp | 23 | All cases in the age group of 2-9 years |
| 7 | Raveendran S 2016 | 2015 | Vadi to Vedikunnu area, Kollam district, Kerala | Coastal | 98 | 6-15 years: 3.2%  16-25 years: 21.4%  26-35 years: 24.5%  36-45 years: 23.4% |
| 8 | Gurav YK 2019 | 2016 | Nellikuzhi, Ernakulam, Kerala | Rural | 562 | 40-68 years: 57/562 (10.1%)  20-39 years: 380/562 (67.6%)  10-19 years: 109/562 (19.4%)  <10 years: 16/562 (2.9%) |
| 9 | Barathidasan R 2020 | 2018 | Vairapuram, Villupuram district, Tamil Nadu | Rural | 26 | 2-5 years: 17/111 (15.3%)  6-10 years: 8/110 (7.3%)  11-15 years: 1/116 (0.9%) |
| 10 | Srinivasan M 2020 | 2019 | Vellore, Tamil Nadu | Semi-urban | 58 | 57 were children aged < 15 years; one adult aged 19 years; <5 years: 8/537 (1.5%)  5-10 years: 28/1005 (2.8%)  10-15 years: 22/938 (2.4%) |

**References**

1. Joshi YK, Tandon BN, Gandhi BM. Hepatitis A epidemic in Kerala state (India) in 1980. Indian J Med Res 1985;81:96-101.
2. Chadha MS, Lole KS, Bora MH, Arankalle VA. Outbreaks of hepatitis A among children in western India. Trans R Soc Trop Med Hyg. 2009;103(9):911-916.
3. Arankalle VA, Sarada Devi KL, Lole KS, et al. Molecular characterization of hepatitis A virus from a large outbreak from Kerala, India. Indian J Med Res. 2006;123(6):760-769.
4. Chobe LP, Arankalle VA. Investigation of a hepatitis A outbreak from Shimla Himachal Pradesh. Indian J Med Res. 2009;130(2):179-184.
5. Rakesh PS, Mainu TTCR, Raj A, et al. Investigating a community wide outbreak of hepatitis A in Kerala, India. J Family Med Prim Care. 2018;7(6):1537-1541.
6. Pal S, Juyal D, Sharma M, et al. An outbreak of hepatitis A virus among children in a flood rescue camp: A post-disaster catastrophe. Indian J Med Microbiol. 2016;34(2):233-236.
7. Raveendran S, Rakesh PS, Dev S, et al. Investigation of an Outbreak of Hepatitis A in a Coastal Area, Kerala, Southern India. J Prim Care Community Health. 2016;7(4):288-290.
8. Gurav YK, Retheesh Babu G, Vinu KP, et al. Suspected spread of hepatitis A virus from a restaurant among adults in rural area of the Kerala state, India. Epidemiol Infect. 2019;147:e210.
9. Barathidasan R, Vanathy K, Venkatesh K, Sharmila FM, Dhodapkar R. Epidemiological and molecular investigation of a hepatitis A outbreak in Tamil Nadu, Southern India. J Infect Dev Ctries. 2020;14(12):1475-1479.
10. Srinivasan M, Sindhu KN, Kumar SJ, et al. Hepatitis A Outbreak with the Concurrence of Salmonella Typhi and Salmonella Poona Infection in Children of Urban Vellore, South India. Am J Trop Med Hyg. 2020;102(6):1249-1252.

**Table 3:** **Studies on seroprevalence of IgG antibodies against HAV in India**

| **R.No** | **Author** | **Year of publication** | **Location** | **Study setting** | **Study population** | **Total samples** | **Laboratory test for IgG** |
| --- | --- | --- | --- | --- | --- | --- | --- |
| 1 | Tandon | 1984 | North India | Community | Children and adults | 483 | HAVAB, Abbott |
| 2 | Werner GT | 1989 | Rural Punjab | NA | Healthy, aged 1-76 years | 385 | HAVAB, Abbott |
| 3 | Graham DY | 1991 | Hyderabad | Hospital | Adults and children attending hospital for minor illness | 238 | HAVAB, Abbott |
| 4 | Arankalle VA | 1995 | Pune | Community | Children and adults | 1982:485  1992: 661 | HAVAB, Abbott |
| 5 | Thapa BR | 1995 | Chandigarh | Schools | Age 3-15 years | 334 | HAVAB, Abbott |
| 6 | Das K | 1996 | Delhi | Hospital | Adults | 95 | NA |
| 7 | Das K | 1998 | Delhi | Hospital | >11 years | 395 | HEPAVASE A-96 |
| 8 | Dhawan PS | 1998 | Mumbai | Hospital | Age <5 years | 670 | IgG ELISA |
| 9 | Mittal SK | 1998 | Delhi | NA | <10 years | 365 | NA |
| 10 | Chadha MS | 1999 | Pune | Hospital | <6 years | 499 | NA |
| 11 | Aggarwal R | 1999 | Lucknow | Hospital | <18 years | 73 | HAVAB, Abbott |
| 12 | Chitambar SD | 1999 | Pune district, Bhor Taluk | Schools | 5-15 years & >16 years | 1983: 252  1987: 327  1995: 402 | ELISA developed by ICMR-NIV |
| 13 | Dutta AK | 2000 | Delhi | Hospital | Age 0-12 years | 420 | Hepvase A 96 TMB |
| 14 | Joshi N | 2000 | Hyderabad | Clinic | Age 2-64 years | 90 | HEPAVASE A-96 |
| 15 | Das K | 2000 | Delhi | Hospital | >15 years | 1580 | HAVAB, Abbott |
| 16 | Murhekar M | 2001 | Andaman & Nicobar | Community | Four tribes | 1989: 240  1999: 814 | ELISA developed by ICMR-NIV |
| 17 | Batra Y | 2002 | Delhi | Schools | 10–17-year-old students | 486 | IgG ELISA |
|  | **Author** | **Year of publication** | **Location** | **Study setting** | **Study population** | **Total samples** | **Laboratory test for IgG** |
| 18 | Jindal M | 2002 | Delhi | Hospital | Medical Students | 91 | IgG ELISA |
| 19 | Xavier S | 2003 | Perintalmanna,Kerala | Hospital | Non-cirrhotic adult patients | 50 | IG ELISA |
| 20 | Mohanvalli B | 2003 | Chennai | School | Age 6 months and 12 years | 185 | IgG ELISA |
| 21 | Acharya SK | 2003 | Delhi | Schools | Age 4-18 years | 1424 | anti-HAV IgG (Organon, Boxtel) |
| 22 | Ramachandran J | 2004 | Vellore | Hospital | Age and sex matched non-CLD controls | 79 | IgG (Genelab diagnostic kits) |
| 23 | Anand AC | 2004 | Delhi | Hospital | Hospital non-CLD cases | 89 | IgG ELISA |
| 24 | Gadgil PS | 2008 | Pune | Hospital | Blood donors | 1145 | IgG ELISA |
| 25 | Rath CP | 2011 | Bijapur | Hospital | Children 6 months-15 years | 142 | Wantai ELISA |
| 26 | Kotwal A | 2014 | Multi-centric | Army trainees | Young adults | 4175 | IgG ELISA |
| 27 | Gupta R | 2019 | North India | Hospital | Children aged 1-5 years | 1084 | IgG ELISA |
| 28 | Chauhan S | 2019 | Lucknow | Hospital | Health care workers | 254 | HAV Ab kit (Dia.Pro, Milan, Italy) |
| 29 | Sood V | 2019 | Delhi | Hospital | Children and adults | 2599 | HAVAB Abbott |
| 30 | Deoshatwar AR | 2020 | Pune | Community | 6-10, 16-25 and 40+ years | 1408 | HEPAVASE A-96 |
| 31 | Agarwal J | 2022 | Lucknow | Hospital | Patients | 1250 | HAV Ab kit (Dia.Pro, Milan, Italy) |
| 32 | Lalwani S | 2025 | Four cities | Hospital | Patients/relatives | 1980 | HEPAVASE A-96 |

**References**

1. Tandon BN, Gandhi BM, Joshi YK. Etiological spectrum of viral hepatitis and prevalence of markers of hepatitis A and B virus infection in north India. Bull World Health Organ 1984; 62: 67-73.
2. Werner GT, Frosner GG, Sareen DK. Prevalence of hepatitis A, B and HIV markers in Punjab. J Indian Med Assoc 1990; 88: 293-294.
3. Graham DY, Adam E, Reddy GT, et al. Seroepidemiology of Helicobacter pylori infection in India. Comparison of developing and developed countries. Dig Dis Sci 1991; 36: 1084-1088.
4. Arankalle VA, Tsarev SA, Chadha MS, et al. Age-specific prevalence of antibodies to hepatitis A and E viruses in Pune, India, 1982 and 1992. J Infect Dis. 1995 Feb;171(2):447-50.
5. Thapa BR, Singh K, Singh V, et al. Pattern of hepatitis A and hepatitis B virus markers in cases of acute sporadic hepatitis and in healthy school children from north west India. J Trop Pediatr 1995; 41: 328-329.
6. Das K, Chakravorty A, Kar P, et al. Seroprevalence of hepatitis A IgG antibody—is it relevant in clinical practice. Indian J Gastroenterol 1996; 15: 32-33.
7. Das K, Kar P, Chakraborty A, et al. Is a vaccination program against hepatitis A needed in India? Indian J Gastroenterol 1998; 17: 158.
8. Dhawan PS, Shah SS, Alvares JF, et al. Seroprevalence of hepatitis A in Mumbai, and immunogenicity and safety of hepatitis A vaccine. Indian J Gastroenterol 1998; 17: 16-18.
9. Mittal SK, Rastogi A, Rastogi A, Kuman N, Talukdar B, Kar P. Seroprevalence of hepatitis A in children – implications for hepatitis A vaccine. Trop Gastroenterol 1998; 19: 120-121.
10. Chadha MS, Chitambar SD, Shaikh NJ, Arankalle VA. Exposure of Indian children to hepatitis A virus & vaccination age. Indian J Med Res 1999; 109: 11-15.
11. Aggarwal R, Naik S, Yachha SK, Naik SR. Seroprevalence of antibodies to hepatitis A virus among children in northern India. Indian Pediatr 1999; 36: 1248-1250.
12. Chitambar SD, Chadha MS, Joshi MS, Arankalle VA. Prevalence of hepatitis A antibodies in western Indian population: changing pattern. Southeast Asian J Trop Med Public Health 1999; 30: 273-276.
13. Dutta AK, Aggarwal A, Kapoor AK, Ray GN, Batra S. Seroepidemiology of hepatitis A in Delhi. Indian J Pediatr 2000; 67: 77-79.
14. Joshi N, Yr NK, Kumar A. Age related seroprevalence of antibodies to hepatitis A virus in Hyderabad, India. Trop Gastroenterol 2000; 21: 63-65.
15. Das K. The changing epidemiological pattern of hepatitis A in an urban population of India: emergence of a trend similar to the European countries. Eur J Epidemiol 2000; 16: 507-510.
16. Murhekar MV, Sehgal SC, Murhekar KM, et al. Changing scenario of hepatitis A virus and hepatitis E virus exposure among the primitive tribes of Andaman and Nicobar Islands, India over the 10-year period 1989-99. J Viral Hepat 2002; 9: 315-321.
17. Batra Y, Bhaktal B, Ojha B, et al. Vaccination against hepatitis A virus may not be required for school children in northern India: results of a sero-epidemiological survey. Bull World Health Organ 2002; 80: 728-731.
18. Jindal M, Rana SS, Gupta RK, Das K, Kar P. Serological study of hepatitis A virus infection amongst the students of a medical college in Delhi and evaluation of the need of vaccination. Indian J Med Res 2002; 115: 1-4.
19. Xavier S, Anish K. Is hepatitis A vaccination necessary in Indian patients with cirrhosis of liver? Indian J Gastroenterol 2003; 22: 54-55.
20. Mohanavalli B, Dhevahi E, Menon T, et al. Prevalence of antibodies to hepatitis A and hepatitis E virus in urban school children in Chennai. Indian Pediatr 2003; 40: 328-331.
21. Acharya SK, Batra Y, Bhaktal B, et al. Seroepidemiology of hepatitis A virus infection among school children in Delhi and north Indian patients with chronic liver disease: implications for HAV vaccination. J Gastroenterol Hepatol 2003; 18: 822-827.
22. Ramachandran J, Eapen CE, Kang G, et al. Hepatitis E superinfection produces severe decompensation in patients with chronic liver disease. J Gastroenterol Hepatol 2004; 19: 134- 138.
23. Anand AC, Nagpal AK, Seth AK, Dhot PS. Should one vaccinate patients with chronic liver disease for hepatitis A virus in India? J Assoc Physicians India 2004; 52: 785-787.
24. Gadgil PS, Fadnis RS, Joshi MS, Rao PS, Chitambar SD. Seroepidemiology of hepatitis A in voluntary blood donors from Pune, western India (2002 and 2004-2005). Epidemiol Infect 2008; 136: 406-409.
25. Rath CP, Akki A, Patil SV, Kalyanshettar SS. Seroprevalence of hepatitis A virus antibody in Bijapur, Karnataka. Indian Pediatr. 2011 Jan;48(1):71-3.
26. Kotwal A, Singh H, Verma AK, et al. A study of Hepatitis A and E virus seropositivity profile amongst young healthy adults in India. Med J Armed Forces India. 2014;70(3):225-229.
27. Gupta R, Sanjeev RK, Agarwal A, et al. A study of hepatitis A virus seropositivity among children aged between 1 and 5 years of age: Implications for universal immunization. Med J Armed Forces India. 2019 Jul;75(3):335-338.
28. Chauhan S, Agarwal J, Jain A, et al. Status of adult immunity to hepatitis A virus in healthcare workers from a tertiary care hospital in north India. Indian J Med Res. 2019 Nov;150(5):508-511.
29. Sood V, Lal BB, Gupta E, et al. Hepatitis A Virus-related Pediatric Liver Disease Burden and its Significance in the Indian Subcontinent. Indian Pediatr. 2019 Sep 15;56(9):741-744.
30. Deoshatwar AR, Gurav YK, Lole KS. Declining trends in Hepatitis A seroprevalence over the past two decades, 1998-2017, in Pune, Western India. Epidemiol Infect. 2020 May 8;148:e121.
31. Agarwal J, Srivastava S, Verma BP, Mehrotra P. Age Group-Specific Assessment of Changing Seroepidemiology of Hepatitis A Virus Infection in North India. Cureus. 2022 Oct 28;14(10):e30792.
32. Lalwani S, Palkar S, S B, Kaur G, et al. Age-stratified prevalence of anti-hepatitis A virus antibodies in four metropolitan Indian cities and recent changes in Pune city. Indian J Gastroenterol. 2025 Mar 7.

**Table 4:** **Results from studies on seroprevalence of IgG antibodies against HAV in India**

| **R.No.** | **Author/year of study** | **Age groups studied** | **Seroprevalence in %** | | | **Endemicity level** |
| --- | --- | --- | --- | --- | --- | --- |
|  |  |  | **<10 years** | **11-15 years** | **15-30 years** |  |
| 4 | Arankalle VA 1982 | >6 months | 6-10 years: 95 | 11-15 years: 99 | 16-35 years: 98 | High |
| 12 | Chitambar SD 1983 | 5-15 years & >16 years | 5-10 years: 97.6 | 11-15 years:100 | >15 years:100 | High |
| 1 | Tandon A 1984 | >10 years & >60 years | 5-10 years: 90 | NA | NA | High |
| 2 | Werner GT 1986 | 1-76 years | 1-9 years: 88 | 10-19 years: 89 | >20 years: 98 | Intermediate |
| 12 | Chitambar SD 1987 | 5-15 years & >16 years | 5-10 years: 96.5 | 11-15 years:100 | >15 years:100 | High |
| 3 | Graham DY 1988 | 3-70 years | 3-21 years: 98.2 | | NA | Couldn’t be classified |
| 16 | Murhekar 1989 | All ages | <10 years: 80 | 11-20: 98.7 | 21-30: 100 | Intermediate |
| 4 | Arankalle VA 1992 | All ages | 6-10 years: 98 | 11-15 years: 99 | 16-35 years: 98 | High |
| 5 | Thapa BR* | 3-15 years | Low socioeconomic: 96 and high socioeconomic: 85 | | NA | Couldn’t be classified |
| 6 | Das K* | Adults | NA | NA | >18 years: 91.6 | Couldn’t be classified |
| 12 | Chitambar SD 1995 | 5-15 years & >16 years | 5-10 years  Rural: 95.1  Urban: 85.1 | 11-15 years:100 | >15 years:100 | Rural: High  Urban: Intermediate |
| 7 | Das K 1996 | >11 years | NA | 11-20 years: 60.2 | 21-30 years: 56.7 | Couldn’t be classified |
| 8 | Dhawan PS 1998 | 6 months -60 years | <5 years: 38 | 11-15 years: 80 | NA | Couldn’t be classified |
| 9 | Mittal SK 1998 | <10 years | 5-10 years: 79.1 | NA | NA | Intermediate |
| 15 | Das K 1998 | >15 years | NA | NA | 15-24: 54.1 & 24-34:58.7 | Couldn’t be classified |
| 10 | Chadha MS 1999 | <6 years | 49-72 months: 90 | NA | NA | High |
| 11 | Aggarwal 1999 | <18 years | 6-10 years: 91 | 11-18 years | NA | High |
| 16 | Murhekar 1999 | All ages | <10 years: 60 | 11-20: 97.6 | 21-30: 98.6 | Intermediate |
| 13 | Dutta AK 2000 | 0-12 years | 9-10 years: 80 | 10-11: 83.3 | NA | Intermediate |
| 14 | Joshi N 2000 | 2-64 years | <10 years: 31 | <15 years: 75 | 18-64 years: 94.4 | Intermediate |
| 17 | Batra Y 2000 | 10-17 years | NA | 10-14 years: 96.6 | 15-17 years: 98.3 | Couldn’t be classified |
| 18 | Jindal M 2002 | >18 years | NA | NA | 18-23 years: 62.6 | Couldn’t be classified |
| 22 | Ramachandran J 2002 | >18 years | NA | NA | >18 years: 100 | Couldn’t be classified |
| **R.No**. | **Author/year of study** | **Age groups studied** | **Seroprevalence in %** | | | **Endemicity level** |
|  |  |  | **<10 years** | **11-15 years** | **15-30 years** |  |
| 23 | Anand AC 2002 | >18 years | NA | NA | >18 years: 94.6 | Couldn’t be classified |
| 19 | Xavier S 2003 | >18 years | NA | NA | >18 years: 100 | Couldn’t be classified |
| 20 | Mohanvalli B 2003 | 6 months -12 years of age | 8-10 years: 83.3 | 11-12 years: 96.9 | NA | Intermediate |
| 21 | Acharya SK 2003 | 4 to 18 years | 8 to 11 years: 91 | 12 to 18 years: 97 | NA | High |
| 24 | Gadgil PS 2002 | 18-25 years | NA | NA | >18 years: 96.5 | Couldn’t be classified |
| 24 | Gadgil PS 2004-2005 | 18-25 years | NA | NA | >18 years: 92.1 | Couldn’t be classified |
| 25 | Rath CP 2011 | 6 months – 15 years | 6 months-5 years: 35.5 | 5-15 years: 54.5 | NA | Intermediate |
| 26 | Kotwal A 2014 | >18 years | NA | NA | >18 years: 92.7% | Couldn’t be classified |
| 27 | Gupta R 2019 | 1-5 years | 1-5 years: 43.5 | NA | NA | Couldn’t be classified |
| 28 | Chauhan S 2019 | >20 years | NA | NA | >20 years: 97.2 | Couldn’t be classified |
| 29 | Sood V 2019 | All age groups | 6-10 years: 78.9 | 10-18 years: 83.8 | 18-30 years: 89.7 | Intermediate |
| 30 | Deoshatwar AR 1998 | 6-10 & 16-25 years | 6-10 years: 30.9 | NA | 16-25 years: 85.9 | Couldn’t be classified |
| 30 | Deoshatwar AR 2007 | 6-10 & 16-25 years | 6-10 years: 40.4 | NA | 16-25 years: 73.9 | Couldn’t be classified |
| 31 | Agarwal J 2022 | >1 to 80 years | 1-10 years:73 | 10-20 years: 83 | 20-30 years: 92.6 | Intermediate |
| 32 | Lalwani S 2025 | 1-17 & 18-40 years | Pune; 6-10 years: 61.4 | 11-15 years:75.7 | 16-25 years: 92.9 | Intermediate |
|  |  |  | Chennai; 6-10 years: 57.0 | 11-15 years: 63.2 | 16-25 years: 92.9 | Intermediate |
|  |  |  | Ludhiana: 6-10 yrs: 68.4 | 11-15 years: 79.2 | 16-25 years: 78.6 | Intermediate |
|  |  |  | Kolkata: 6-10 yrs: 35.7 | 11-15 years: 43.4 | 16-25 years: 61.9 | Low |

*Year of study was not available

**Supplementary methods: Sample size and sampling methods of COVID-19 serosurvey**

A sample size of 5,929 (rounded to 6,000) was calculated per stratum of districts (districts were categorized into four strata based on the reported COVID-19 incidence) to estimate one per cent seropositivity, with 40 per cent relative precision, 95 per cent confidence interval (CI) and design effect of 2·5. Four hundred individuals were selected from each district. In each district, 10 clusters (village in rural areas and ward in urban areas) were selected by probability proportion to population size. In each cluster, four random locations were selected. A random starting point was selected from each location and all contiguous households were visited until 10 eligible individuals were enrolled.

Reference: Murhekar MV, Bhatnagar T, Selvaraju S, et al. Prevalence of SARS-CoV-2 infection in India: Findings from the national serosurvey, May-June 2020. Indian J Med Res 2020;152:48-60.

**Design weight calculation:**

We calculated the probability of selection of the sampling unit at each of the two stages of selection (districts, villages/wards). The compound probability was obtained by the product of the probabilities of the two stages. We calculated the design weight by taking the inverse of the compound probability. We then calculated normalised standard design weights. We then analysed the complex sample clustered data with normalized design weights to get the seroprevalence and 95 percent CI using the survey data analysis module in the STATA software.

**Table 5:** **Quality assurance of laboratory testing**

| **Results of initial test** | **Results of repeat test** | | **Total** |
| --- | --- | --- | --- |
|  | Nonreactive | Reactive |  |
| Nonreactive | 118 | 17 | 135 |
| Reactive | 7 | 604 | 611 |
| **Total** | 125 | 621 | 746 |

**Figure: Age-specific seroprevalence curve by state**


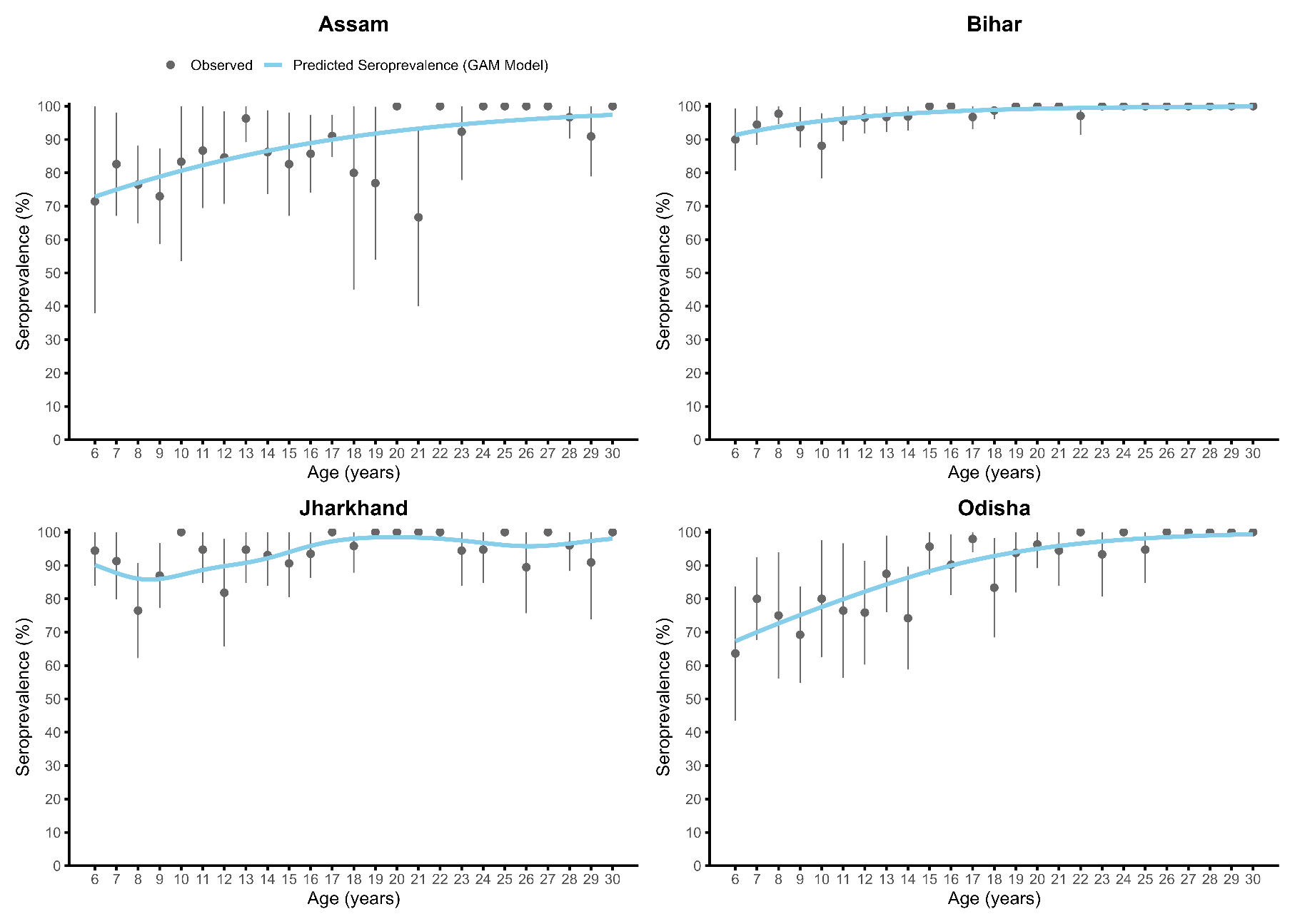


**Figure: Age-specific seroprevalence curve by state**


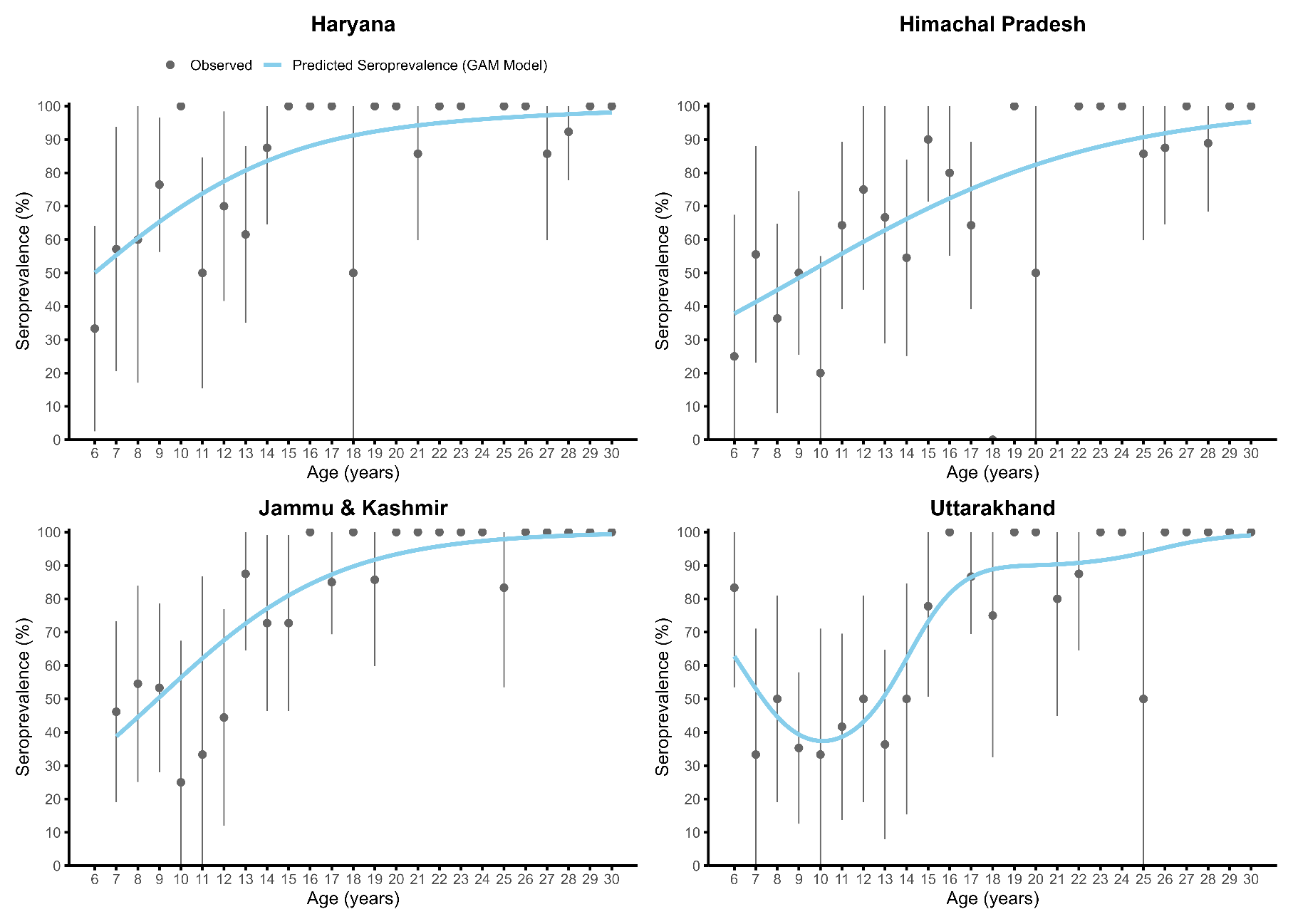


**Figure: Age-specific seroprevalence curve by state**


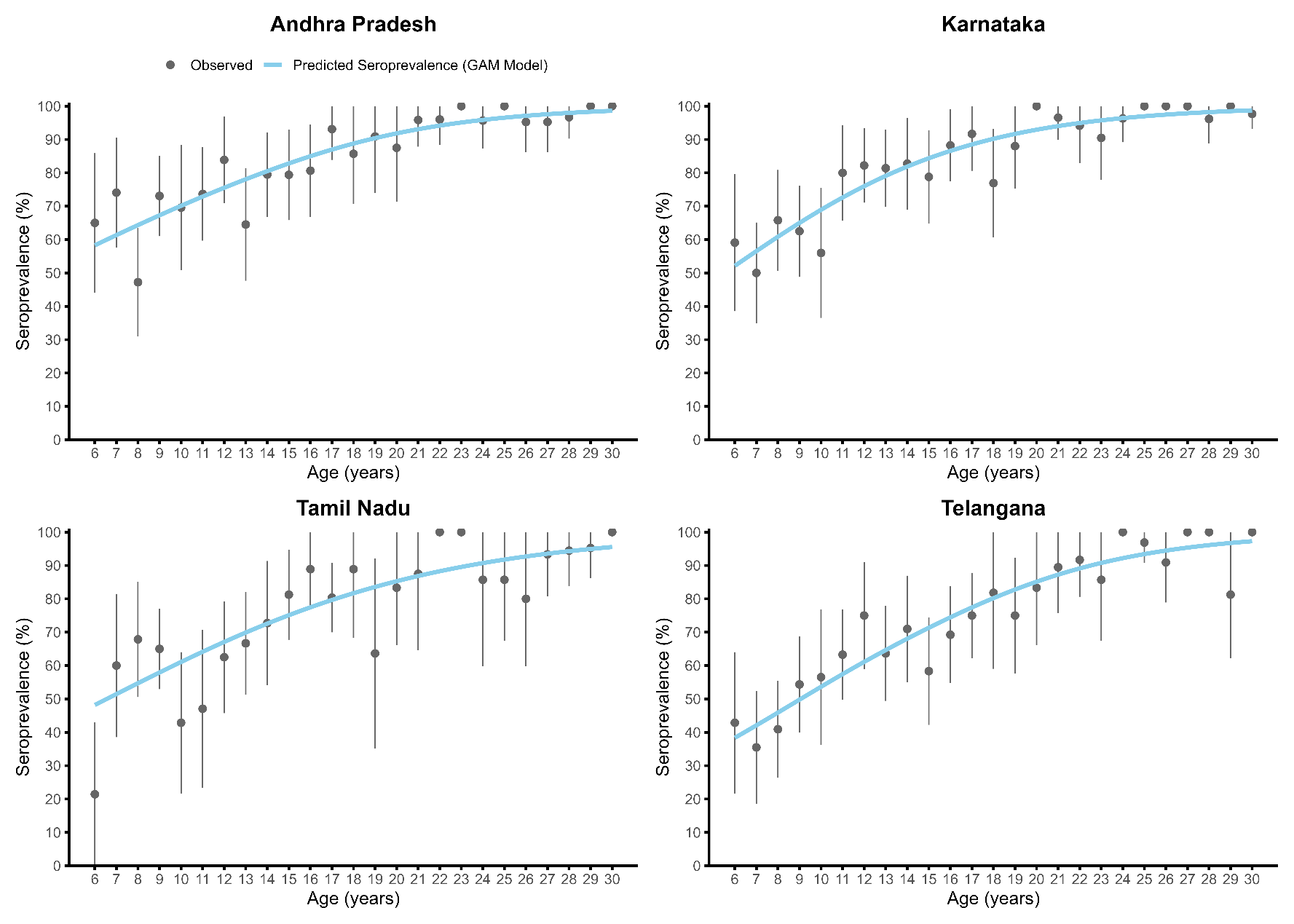


**Figure: Age-specific seroprevalence curve by state**


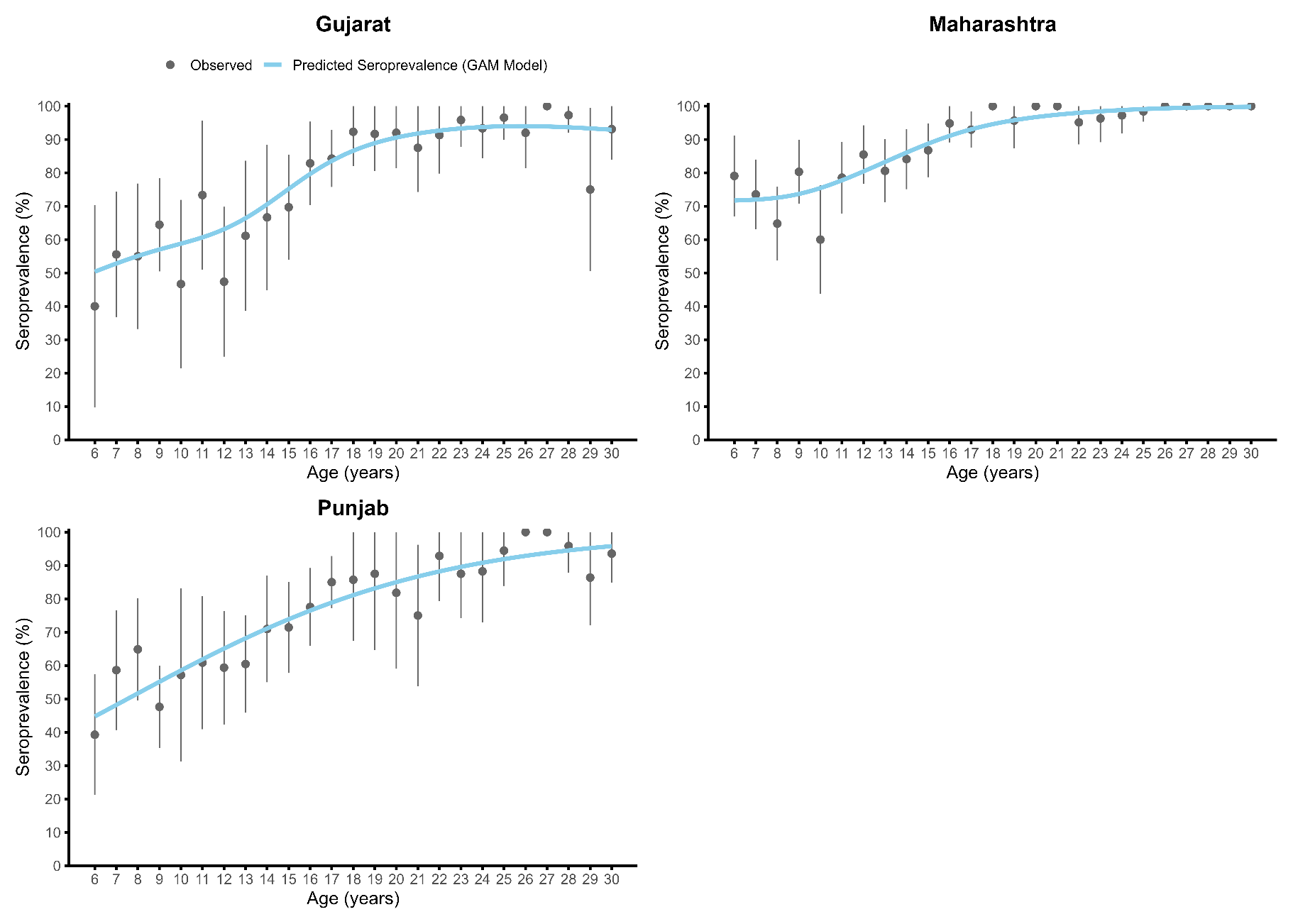


**Figure: Age-specific seroprevalence curve by state**


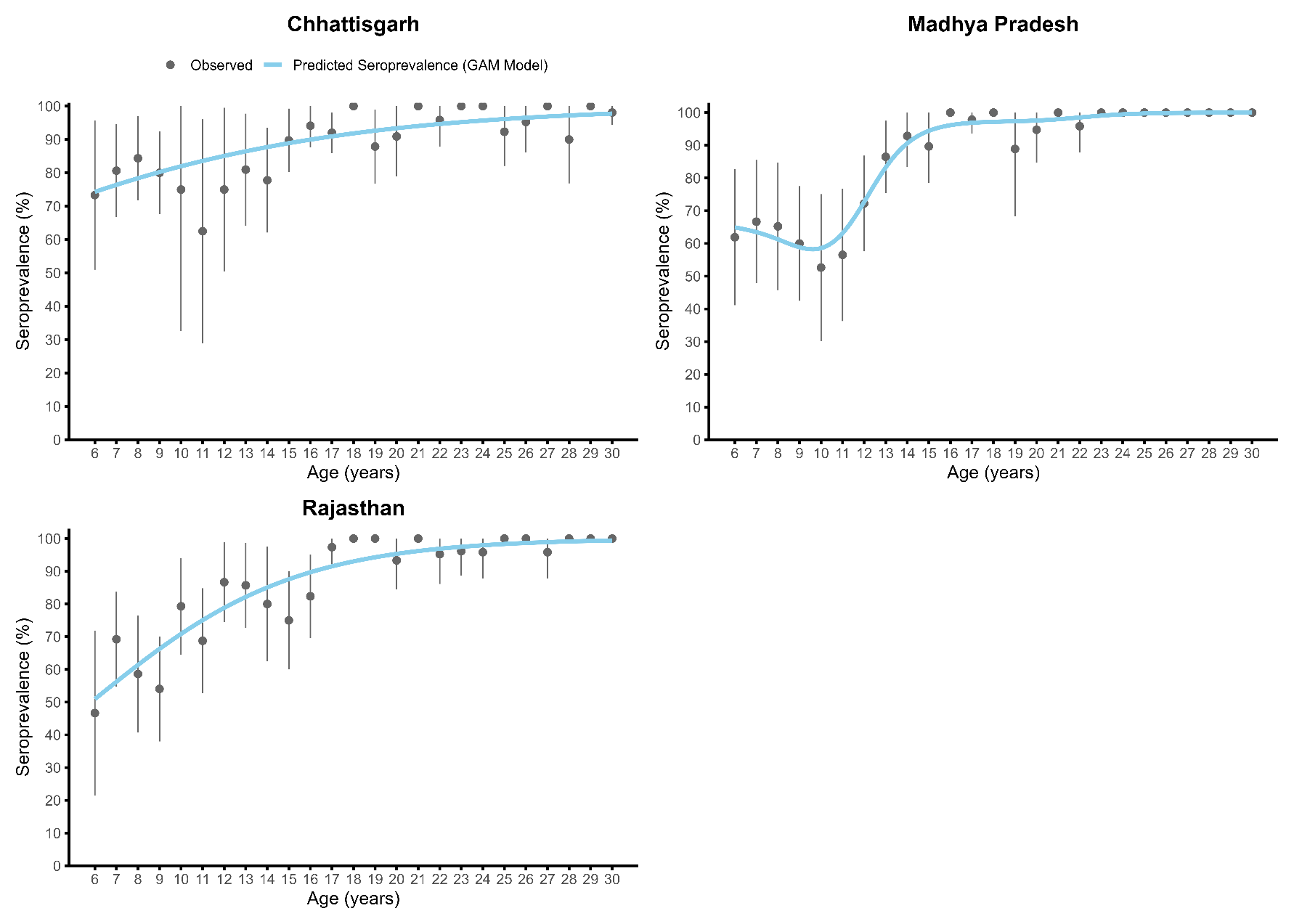


**Table 6: Findings from studies that tested acute liver failure cases for IgM antibodies against hepatitis A virus in India**

| **R.No** | **Author/Year of publication** | **Age group** | **Location** | **# ALF cases included (N)** | **# ALF cases tested positive for anti-HAV IgM (n)** | **% positive for HAV (n/N*100)** | **Remarks** |
| --- | --- | --- | --- | --- | --- | --- | --- |
| **1** | Samantha T 2007 | 1-12 years | Kolkata, West Bengal | 45 | 16 | 35.5 | HAV and HEV were the commonest |
| 2 | Pandit A 2015 | 0-18 years | Vellore, Tamil Nadu and Pune, Maharashtra | 41 | 27 | 65.9 | Of the 27 cases, 6 died. HAV and HBV were most common |
| 3 | Das AK 2016 | >=14 years | Dibrugarh, Assam | 255 | 76 | 29.8 | HAV and HEV were the commonest |
| 4 | Mallick S 2020 | All ages | Kochi, Kerala | 70 | 15 | 21.4 | Yellow phosphorous poisoning was the most commonest |
| 5 | Seth S 2024 | Adults | Jaipur, Rajasthan | 32 | 8 | 25.0 | HBV is the next commonest cause |
| 6 | Roy A 2025 | >18 years | Chandigarh, Kolkata, Jaipur, Hyderabad | 183 | 81 | 44.2 | Rodenticide poisoning was the next commonest |

**References**

1. Samanta T, Ganguly S. Aetiology, clinical profile and prognostic indicators for children with acute liver failure admitted in a teaching hospital in Kolkata. Trop Gastroenterol 2007 ;28(3):135-9.
2. Pandit A, Mathew LG, Bavdekar A, et al. Hepatotropic viruses as etiological agents of acute liver failure and related-outcomes among children in India: a retrospective hospital-based study. BMC Res Notes 2015 ;8:381.
3. Das AK, Begum T, Kar P, Dutta A. Profile of Acute Liver Failure from North-east India and Its Differences from other Parts of the Country. Euroasian J Hepatogastroenterol 2016 ;6(2):111-115.
4. Mallick S, Nair K, Thillai M, et al. Liver Transplant in Acute Liver Failure - Looking Back Over 10 Years. J Clin Exp Hepatol 2020;10(4):322-328.
5. Seth S, Maharshi S, Sharma KK, Pokharna R, Nijhawan S, Sharma SS. Changing etiological spectrum of acute liver failure. Indian J Gastroenterol 2024;43(2):452-458.
6. Roy A, Kumar K, Premkumar M, et al. Current status of etiology and outcomes of acute liver failure in India-A multicentre study from tertiary centres. Indian J Gastroenterol 2025;44(1):47-56.

**Table 7:** **National Family Health Survey data on sanitation and Human Development Index (HDI) score**

|  | % households with any toilet or latrine facility | | | | | HDI score (2017-2018) | HAV seroprevalence (6-30 years) |
| --- | --- | --- | --- | --- | --- | --- | --- |
|  | NFHS-1 | NFHS-2 | NFHS-3 | NFHS-4 | NFHS-5 |  |  |
| Kerala | 70.9 | 85.2 | 96.1 | 99.2 | 99.7 | 0.775 | 44.8 |
| West Bengal | 40.4 | 44.8 | 59.6 | 74.9 | 88.0 | 0.674 | 63.1 |
| Punjab | 36.7 | 51.4 | 70.8 | 92.9 | 97.2 | 0.738 | 69.7 |
| Uttarakhand | NA | NA | NA | NA | 93.6 | 0.758 | 70.6 |
| Himachal Pradesh | 12.6 | 26.7 | 46.4 | 85.7 | 93.5 | 0.761 | 70.8 |
| Jammu and Kashmir | 19.1 | 51.1 | 61.7 | 79.3 | 94.3 | 0.663 | 74.6 |
| Telangana | NA | NA | NA | 69.0 | 87.3 | 0.705 | 74.8 |
| Tamil Nadu | 29.4 | 34.0 | 42.9 | 61.7 | 77.5 | 0.738 | 75.1 |
| Andhra Pradesh | 24.4 | 27.3 | 42.4 | 61.3 | 83.7 | 0.648 | 76.7 |
| Gujarat | 35.8 | 44.9 | 54.6 | 71.0 | 80.7 | 0.698 | 81.0 |
| Haryana | 26.9 | 39.0 | 52.4 | 89.8 | 96.6 | 0.724 | 82.8 |
| Karnataka | 31.2 | 38.6 | 46.5 | 65.8 | 82.3 | 0.706 | 83.1 |
| Rajasthan | 19.8 | 27.8 | 30.8 | 54.0 | 77.5 | 0.638 | 86.0 |
| Chhattisgarh | NA | NA | 18.7 | 41.3 | 84.7 | 0.629 | 86.9 |
| Odisha | 12.2 | 13.5 | 19.3 | 35.0 | 66.1 | 0.649 | 87.9 |
| Assam | 49.6 | 63.0 | 76.4 | 88.9 | 95.8 | 0.651 | 88.5 |
| Maharashtra | 40.8 | 45.9 | 52.9 | 71.2 | 82.6 | 0.750 | 90.4 |
| Madhya Pradesh | 21.3 | 22.2 | 27.0 | 45.8 | 73.8 | 0.616 | 90.7 |
| Jharkhand | NA | NA | 22.6 | 30.0 | 66.4 | 0.618 | 95.2 |
| Bihar | 16.5 | 16.8 | 25.2 | 33.5 | 61.1 | 0.551 | 96.1 |
| Uttar Pradesh | 22.9 | 26.5 | 33.1 | 45.8 | 77.1 | 0.592 | 97.0 |
| India | 30.3 | 35.9 | 44.6 | 61.1 | 80.6 | 0.672 |  |

**Table 8: Comparison of demographic characteristics of the study population with the 2011 census data**

| **Characteristics** | **N=14778**  **n (%)** | **Census 2011**  **%** |
| --- | --- | --- |
| **Age in years** |  |  |
| 6 - 10 | 3266 (22.1) | 22 |
| 11-15 | 3357 (22.7) | 21 |
| 16-30 | 8155 (55.2) | 57 |
| **Gender** |  |  |
| Male | 7267 (49.2) | 51.5 |
| Female | 7492 (50.7) | 48.5 |
| Others | 19 (0.13) |  |
| **Area of residence** |  |  |
| Rural | 11230 (76.0) | 68.8 |
| Urban | 3548 (24.0) | 31.2 |

**Strengthening the Reporting of Observational Studies in Epidemiology (STROBE) checklist**

|  | Item No | Recommendation | Location in the manuscript |
| --- | --- | --- | --- |
| **Title and abstract** | 1 | (*a*) Indicate the study’s design with a commonly used term in the title or the abstract | Page 1 (Ln 1,2) Page 2 (Ln 53) |
|  |  | (*b*) Provide in the abstract an informative and balanced summary of what was done and what was found | Page 2 |
| Introduction | | | |
| Background/rationale | 2 | Explain the scientific background and rationale for the investigation being reported | Page 4,5 |
| Objectives | 3 | State specific objectives, including any prespecified hypotheses | Page 5 (Ln 166-168) |
| Methods | | | |
| Study design | 4 | Present key elements of study design early in the paper | Page 6 (Ln 171) |
| Setting | 5 | Describe the setting, locations, and relevant dates, including periods of recruitment, exposure, follow-up, and data collection | Page 6 (Ln 172-191) |
| Participants | 6 | (*a*) Give the eligibility criteria, and the sources and methods of selection of participants | Ln 178-179 |
| Variables | 7 | Clearly define all outcomes, exposures, predictors, potential confounders, and effect modifiers. Give diagnostic criteria, if applicable | NA |
| Data sources/ measurement | 8* | For each variable of interest, give sources of data and details of methods of assessment (measurement). Describe comparability of assessment methods if there is more than one group | Page 6, Ln 193-199 |
| Bias | 9 | Describe any efforts to address potential sources of bias | Page 7, Ln 200-201 |
| Study size | 10 | Explain how the study size was arrived at | Page 6, Ln 173-179 |
| Quantitative variables | 11 | Explain how quantitative variables were handled in the analyses. If applicable, describe which groupings were chosen and why | Ln 198-199 |
| Statistical methods | 12 | (*a*) Describe all statistical methods, including those used to control for confounding | Page 7, Ln 203-215 |
|  |  | (*b*) Describe any methods used to examine subgroups and interactions | Ln 206-207 |
|  |  | (*c*) Explain how missing data were addressed | NA |
|  |  | (*d*) If applicable, describe analytical methods taking account of sampling strategy | Ln 204-205 |
|  |  | (*e*) Describe any sensitivity analyses | NA |
| Results | | | |
| Participants | 13* | (a) Report numbers of individuals at each stage of study—eg numbers potentially eligible, examined for eligibility, confirmed eligible, included in the study, completing follow-up, and analysed | Figure 1, Ln 220-224 |
|  |  | (b) Give reasons for non-participation at each stage | Figure 1 |
|  |  | (c) Consider use of a flow diagram | Figure 1 |
| Descriptive data | 14* | (a) Give characteristics of study participants (eg demographic, clinical, social) and information on exposures and potential confounders | Ln 224-228 |
|  |  | (b) Indicate number of participants with missing data for each variable of interest | NA |
| Outcome data | 15* | Report numbers of outcome events or summary measures | Page 8, Ln 229-232 |
| Main results | 16 | (*a*) Give unadjusted estimates and, if applicable, confounder-adjusted estimates and their precision (eg, 95% confidence interval). Make clear which confounders were adjusted for and why they were included | Table 2 |
|  |  | (*b*) Report category boundaries when continuous variables were categorized | NA |
|  |  | (*c*) If relevant, consider translating estimates of relative risk into absolute risk for a meaningful time period | NA |
| Other analyses | 17 | Report other analyses done—eg analyses of subgroups and interactions, and sensitivity analyses | Ln 245-247 |
| Discussion | | | |
| Key results | 18 | Summarise key results with reference to study objectives | Ln 257-262 |
| Limitations | 19 | Discuss limitations of the study, taking into account sources of potential bias or imprecision. Discuss both direction and magnitude of any potential bias | Ln 328-337 |
| Interpretation | 20 | Give a cautious overall interpretation of results considering objectives, limitations, multiplicity of analyses, results from similar studies, and other relevant evidence | Ln 263-275 |
| Generalisability | 21 | Discuss the generalisability (external validity) of the study results | Ln 276-292 |
|  | | | |
| Funding | 22 | Give the source of funding and the role of the funders for the present study and, if applicable, for the original study on which the present article is based | Ln 217-218 |

*Give information separately for exposed and unexposed groups.
